# Feasibility of integrating Cultural Consensus Modeling into community-engaged research: lessons from rural Arizona

**DOI:** 10.3389/fpubh.2026.1741925

**Published:** 2026-08-21

**Authors:** Mark Remiker, Caroline Mende, Alexandra Olin, Melissa A. Liebert, Michael Nugent, Nick Calles, Amanda Aguirre, Marisol Penuelas-Arrizon, Janelle Linn, Camilla Adams, Regina Eddie, Nicolette I. Teufel-Shone, Samantha Sabo

**Affiliations:** 1 Center for Community Health and Engaged Research, Northern Arizona University, Flagstaff, AZ, United States; 2 Department of Anthropology, Northern Arizona University, Flagstaff, AZ, United States; 3 Regional Center for Border Health, Inc., Somerton, AZ, United States; 4 Navajo County Public Health Services District, Show Low, AZ, United States; 5 Canyonlands Healthcare, Page, AZ, United States; 6 College of Nursing, Northern Arizona University, Flagstaff, AZ, United States; 7 Department of Health Sciences, Northern Arizona University, Flagstaff, AZ, United States

**Keywords:** community health priorities, community-engaged research, Cultural Consensus Modeling, Research Centers in Minority Institutions, survey development

## Abstract

This study examines the feasibility of integrating Cultural Consensus Modeling (CCM)—a structured anthropological method for identifying shared cultural beliefs—into community-engaged public health research within the Research Centers in Minority Institutions (RCMI) framework. In collaboration with the RCMI Community Expert Board at Northern Arizona University, researchers developed a mixed-methods questionnaire to identify community assets, challenges, and health priorities across rural, border, and underserved communities in Arizona. Guided by the Vitalyst Health Foundation’s “Elements of a Healthy Community” framework, the instrument was co-developed and pilot-tested in English and Spanish. Over a six-month period, 261 participants from four congressional districts and six counties completed the survey, supported by recruitment efforts from three regional partner organizations. Post-recruitment interviews with community partners revealed key implementation challenges, including digital literacy barriers and trust in external institutions, as well as recommendations for future multimodal survey dissemination. Findings demonstrate the feasibility and value of using CCM to systematically capture culturally grounded health priorities, offering a promising complement to participatory methods and a pathway for advancing equity-centered, community-defined public health interventions within RCMI initiatives. Because the purpose of this manuscript is to document the feasibility and process of integrating CCM within a community-engaged framework, analytic CCM results are intentionally not included and will be presented in a separate manuscript.

## Introduction

The Research Centers in Minority Institutions (RCMI) program, which is funded by the National Institute on Minority Health and Health Disparities (NIMHD), works to strengthen the research infrastructure at minority-serving institutions. The main goals of the RCMI program are to enhance research capacity, foster the development of the scientific workforce, and address health disparities by improving community health through research (1). An RCMI Community Engagement Core (CEC) ensures that research is truly responsive to community needs by grounding the Center’s work in lived experience and shared priority-setting. Through sustained relationships, transparent communication, and meaningful community participation, the CEC builds trust and ensures that research questions, methods, and dissemination strategies reflect local context and cultural relevance. Specifically, the CEC is tasked with identifying community health priorities to ensure that the scientific goals of the program are grounded in real-world needs of local communities.

When evaluating community-health priorities, traditional top-down models often fail to capture the nuanced, culturally grounded concerns of diverse populations, particularly in rural, underserved, and border regions (2, 3). As a result, public health interventions may lack relevance, effectiveness, and community buy-in. To address these limitations, participatory methodologies such as Community-Based Participatory Research (CBPR) and Participatory Action Research (PAR) have gained traction, emphasizing collaboration between researchers and community members to co-create knowledge and solutions (4). Specifically, these approaches can enhance research by reducing community members’ general distrust in academia and by enabling logistically appropriate data collection methods (5).

Despite the growing use of participatory methods, there remains a gap in tools that can systematically measure shared beliefs and priorities within communities. Cultural Consensus Modeling (CCM), an anthropological technique developed by Romney et al. (6), offers a promising complement to existing participatory methodologies. Notably, CCM is a structured method for identifying the degree of agreement among individuals within a cultural group, allowing researchers to determine whether a shared cultural model exists and who best represents it. Accordingly, integrating CCM with other participatory action methods can help to inform policy by systematically identifying culturally grounded health priorities as opposed to relying on mainstream models that may overlook perspectives from diverse communities and perpetuate inequities (7).

While CCM has been widely applied in anthropology to better understand, for example, how cultural ideals relate to health and conservation behaviors (8–10), its use in public health research remains limited (11, 12). This underutilization among public health professionals may stem from a lack of awareness or training in CCM (13). Additionally, the perceived methodological complexity may deter researchers from using CCM in large-scale public health studies (14). In spite of this, the National Institutes of Health (NIH) have encouraged researchers to use tools like CCM to help to define cultural domains relevant to health and integrate these insights into program design and evaluation (15).

To address these gaps, the present study demonstrates the potential of CCM to enhance culturally competent, community-driven public health efforts, specifically within the RCMI framework. Here, we describe how the Southwest Health Engagement and Research Collaborative (SHERC), an RCMI within Northern Arizona University, integrated CCM with other community-engaged methods to identify local community health priorities across diverse communities in Arizona, with the following main objectives:

1. Provide an overview of the community-engaged, mixed-methods questionnaire development processes aimed at identifying community health priorities.
2. Describe recruitment methods used at each community partner site to enhance participation.
3. Review preliminary questionnaire results and feedback from community partners on their experience collaborating with the SHERC CEC.
4. Discuss potential strengths and challenges of using CCM in public health research.

The purpose of this manuscript is to describe the feasibility and process of integrating Cultural Consensus Modeling into a community-engaged research framework. Consistent with this focus, we do not present CCM analytic results (e.g., consensus estimates, factor structures, competence scores). These findings will be reported in a separate manuscript to allow for adequate depth and clarity.

## Methods

### Cultural Consensus Modeling

CCM is a methodological framework used primarily in anthropology and social sciences to infer shared cultural beliefs or knowledge within a group (6). In addition to identifying shared beliefs, CCM can be used to determine the degree to which individuals agree on culturally relevant information and can reveal intracultural variation by helping researchers identify subgroups with differing views.

CCM uses individual responses from a set of questions, typically about beliefs, practices, or knowledge, and applies them to a factor-analytic model to identify patterns of agreement. Accordingly, if a group shares a common cultural model, then individuals who are more knowledgeable (i.e., have responses closer to the consensus) will agree more with others. Here, we will focus on the process of working with community partners to develop and disseminate a CCM questionnaire. Accordingly, this section describes procedural integration and feasibility considerations rather than the analytic outcomes of the consensus model. Results from the CCM will be presented in a future publications.

### Instrument development

To meet the goals of the SHERC CEC, a mixed-methods questionnaire was developed to identify current community assets and challenges and to define community health priorities in rural, border, and underserved communities in Arizona. The initial draft of the questionnaire was divided into three main sections: 1. Community assets and desired changes (combination of open and closed-ended questions), 2. Community health priorities (Likert-type scale), and 3. Participant demographics.

**Figure 1:**
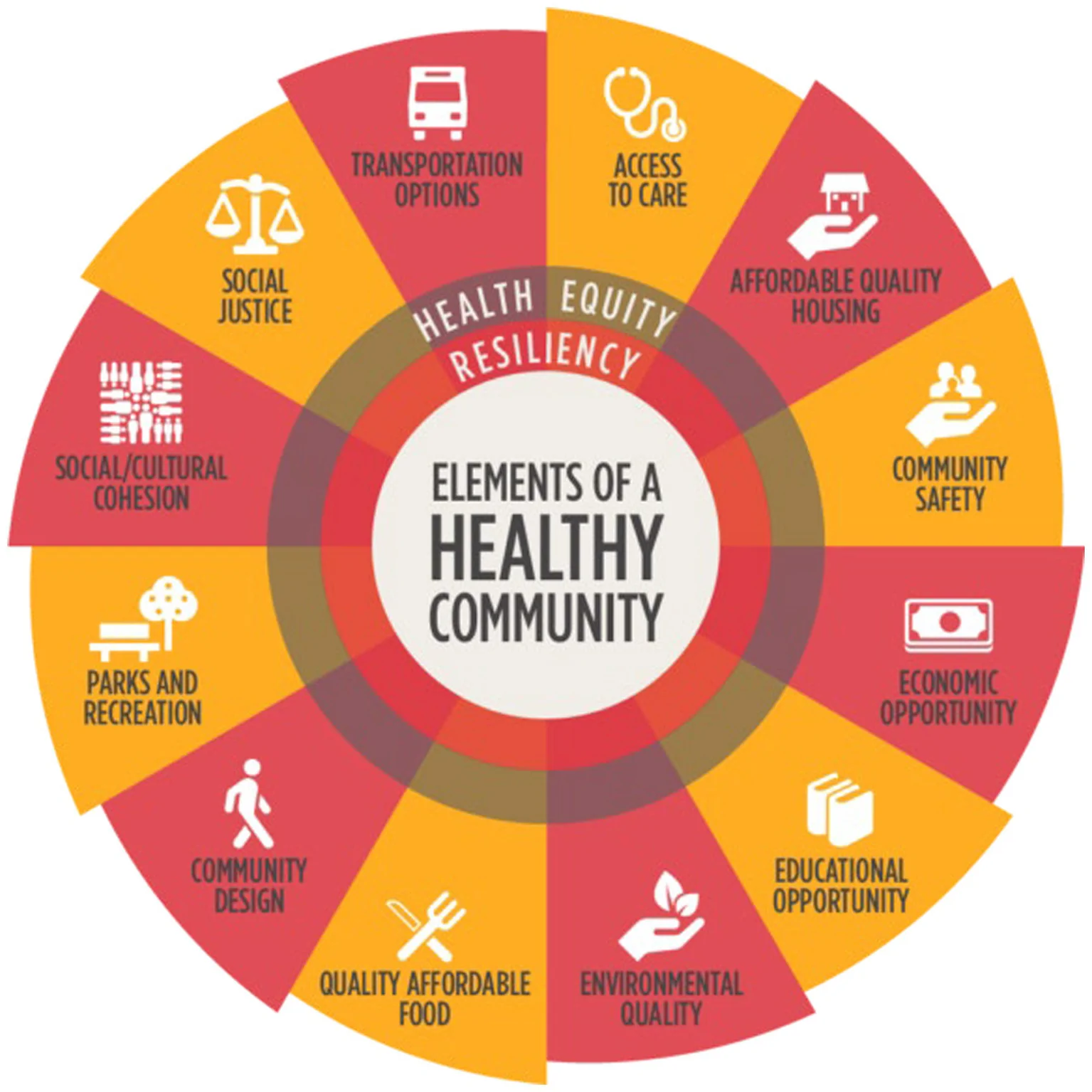
Vitalyst Health Foundation Elements of a Healthy Community Wheel. Reproduced with permission from “The Elements of a Healthy Community wheel”, Vitalyst Health Foundation (www.vitalysthealth.org).

Typically, in the first phase of a CCM study, participants take part in an elicitation technique called free listing (16, 17) to generate lists of items related to a specific domain, like “community health priorities.” The free lists are then analyzed to find the most frequently mentioned (i.e., salient) items, which inform future questionnaire development. In this case, however, the SHERC CEC was fortunate to build off previous work conducted by the Vitalyst Health Foundation (Vitalyst), a public foundation aimed at addressing social determinants of health to improve wellbeing in Arizona. In collaboration with community partners, Vitalyst developed the “Elements of a Healthy Community Wheel” (see Figure 1) (18), which illustrates the major factors that contribute to individual health and more broadly to the overall health of communities in Arizona. Guided by Vitalyst’s work, the SHERC CEC created a set of Likert-type questions that ask participants to rate how important each item is for the health and wellbeing of their community (1 = not important to 5 = essential).

The initial draft of the questionnaire was revised for length and clarity based on feedback from the SHERC Community Expert Board. A final draft of the questionnaire included a total of 58 items across three sections: 1. Community assets and desired changes (eight items), 2. Community health priorities (37 items), and 3. Participant demographics (13 items). Forward translation was used to develop a Spanish version of the questionnaire. Both English and Spanish versions were developed using Qualtrics (19) and pilot tested by members of the community.

### Sampling strategy

With a primary goal of understanding the needs of Arizona’s rural, border, and underserved communities, the SHERC CEC focused their recruitment efforts on four congressional districts (2, 6, 7, and 9) that account for a majority of Arizona’s rural and border population. Additionally, purposive sampling was used to identify communities within those districts that have a high score (above 5) on the Area Deprivation Index (ADI) (20). The ADI is a measure of socioeconomic deprivation based on factors including income, education, employment, and housing quality. A higher ADI score indicates a higher level of socioeconomic disadvantage in a neighborhood. Combining region and ADI score allowed researchers to use targeted recruitment strategies and obtain an applicant pool representative of rural, border, and underserved communities.

Inclusion and exclusion criteria for participants were as follows: 1. Participants must be 18 years or older; 2. Participants must have their primary residence in Arizona, although they were not required to be permanent or legal residents; 3. Participants must reside in the sampling communities identified by the researchers; 4. Participants must have an email address and access to a smartphone or computer; and 5. Due to IRB restrictions, any individuals whose primary residence is located on Native lands were not eligible to participate.

### Community partners

In line with community-engaged research practices (21), the SHERC CEC established partnerships with three trusted community organizations to assist with recruitment efforts. Each partner organization was provided with a $500 stipend to offset administrative burden. Community partner organizations included:

The Regional Center for Border Health, Inc. (RCBH): A 501 (c) 3 non-profit organization established in 1986 whose mission has centered on improving the quality of life of residents along the U.S.–Mexico border by increasing access to quality training and affordable healthcare.

Canyonlands Healthcare (Canyonlands): A Federally Qualified Health Center that provides comprehensive and cost-effective healthcare services to 10 locations in eight rural communities in Arizona and Nevada.

Navajo County Public Health Services District (NCPHSD): A public health department that provides services related to primary prevention, screening, and treatment of diseases and conditions to the residents of Navajo County—a largely rural community located in northeastern Arizona.

### Procedure

Each community partner organization was supplied with flyers that included a link and QR code to the screening survey. In line with a community engaged research approach (22), community partners led all participant recruitment activities using methods amenable to their organizational cultures. For example, RCBH and Canyonlands utilized their Promotoras de salud and Community Health Workers, respectively, to disseminate flyers, while NCPHSD focused their recruitment efforts on clinic settings, including WIC clinics and immunization sites. Additionally, community partners distributed flyers through internal listservs and at community events.

Following the initial screening questions, SHERC CEC staff completed a second round of screening by reviewing Qualtrics data for duplicate IP addresses, IP addresses out of region, and duplicate or similar email addresses. If individuals met all eligibility criteria, then they were sent a personal link to the full questionnaire. Once questionnaires were completed, participants were sent an electronic gift card for $50 through the Tango website which allows recipients to choose from a wide selection of electronic gift cards from different brands. This project was given exempt approval by the Northern Arizona University Institutional Review Board (Project number: 2139514-4), and all participants provided informed consent prior to taking the questionnaire.

### Analytical approach

All descriptive statistics were analyzed using IBM SPSS (Version 30) (23). Qualitative data from open-ended questions were analyzed using *a priori* coding and a thematic analysis approach in ATLAS.ti (Version 9) (24). *A priori* codes were based on the Vitalyst’s Elements of a Healthy Community (18) and subcodes were grounded in participant responses. Emergent coding was used in instances where no *A priori* code existed. Two researchers independently applied the codebook to the same transcripts, wrote brief analytic memos to document their rationale, and then compared coded segments to identify areas of agreement and discrepancy. They met to discuss each difference using code definitions, inclusion/exclusion criteria, and textual evidence to reach consensus and refine the codebook as needed. This iterative code–compare–consensus cycle continued until coding stability was achieved, producing a finalized, consensus-coded dataset for analysis.

Follow up interviews were conducted with each partner organization to determine how recruitment took place at each agency, if difficulties and/or barriers in survey dissemination and participant recruitment were perceived, if additional burdens were placed on staff at each agency, and if the representative had any recommendations for future collaborative projects.

## Results

Because the purpose of this manuscript is to evaluate feasibility and document implementation processes, the results presented below focus on recruitment, participant characteristics, and community partner feedback. Analytic CCM results—including consensus estimates, factor loadings, and competence scores—will be reported in a separate manuscript.

### Participant recruitment

Participant recruitment took place over 6 months (January 2025–June 2025). A total of 741 individuals were screened with 62% (*n* = 463) screening eligible for participation. Of those who were ineligible (*n* = 278), the most common reasons included duplicate or similar email addresses paired with a duplicate IP address (*n* = 85, 31%), primary residence on Tribal lands (*n* = 33, 12%), incomplete screener (*n* = 26, 9%), and IP address out of region (*n* = 22, 8%). Of the 463 eligible participants, 261 completed the survey (English version, *n* = 215; Spanish version, *n* = 46) and were enrolled in the study.

### Participant demographics

Participants were recruited from underserved rural and border communities spanning four Arizona congressional districts (Districts 2, 6, 7, and 9), 6 counties (Coconino, Graham, Greenlee, Navajo, Mohave, and Yuma), and over 25 cities and towns. Table 1 provides a summary of participant demographics by congressional district. The average age of participants was 38 years old (SD = 13.6). Most participants identified as women (*n* = 197, 75%), Latino/Hispanic (*n* = 172, 66%), and White (*n* = 180, 68%), with a relatively equal distribution across political affiliation (Republican: *n* = 86, 33%; Democrat: *n* = 63, 24%; Independent: *n* = 49, 19%).

**Table 1:** Participant demographics.

District	Arizona congressional district				
	2 (*n* = 84)	6 (*n* = 27)	7 (*n* = 131)	9 (*n* = 19)	Total (*N* = 261)
Gender (*n* = 261)					
Female	60 (71%)	21 (78%)	102 (78%)	14 (74%)	197 (75%)
Male	23 (27%)	6 (22%)	27 (20%)	4 (21%)	60 (23%)
Non-binary	0 (0%)	0 (0%)	1 (0.7%)	0 (0%)	1 (0.4%)
No Answer	1 (1%)	0 (0%)	1 (0.7%)	1 (5%)	3 (1.1%)
Race and ethnicity (*n* = 261)					
American Indian/Alaskan Native	20 (24%)	4 (15%)	0 (0%)	1 (5%)	25 (9%)
Black/African American	0 (0%)	1 (4%)	0 (0%)	0 (0%)	1 (0.3%)
Hispanic/Latino	20 (24%)	9 (33%)	129 (98%)	14 (74%)	172 (66%)
White	57 (68%)	23 (85%)	84 (64%)	16 (84%)	180 (69%)
No answer	5 (6%)	0 (0%)	9 (7%)	0 (0%)	14 (5%)
Age in years (*n* = 258)					
Mean (SD)	40.7 (13.6)	35.9 (11.5)	35.3 (13.2)	44.9 (15.3)	38 (13.6)
Political affiliation (*n* = 259)					
Republican	43 (51%)	15 (56%)	23 (19%)	5 (26%)	86 (33%)
Democrat	13 (15%)	6 (22%)	43 (33%)	1 (5%)	63 (24%)
Independent	19 (23%)	4 (15%)	19 (15%)	7 (37%)	49 (19%)
Libertarian	0 (0%)	0 (0%)	4 (3%)	0 (0%)	4 (1.5%)
Other	8 (10%)	2 (7%)	41 (31%)	6 (32%)	57 (22%)

Most participants responded that they felt a part of their community (*n* = 227, 87%), and over 60% (*n* = 161) had lived in their community for over 10 years. Half of all participants were married (*n* = 130), and approximately two-thirds (*n* = 171) reported having full time employment; however, household income ranged between less than $15,000 and over $75,000 annually. Self-reported job titles included electrician, medical assistant, roofer, dental hygienist, legal assistant, waitress, registered nurse, and welder, to name a few.

### Community health priorities

**Figure 2:**
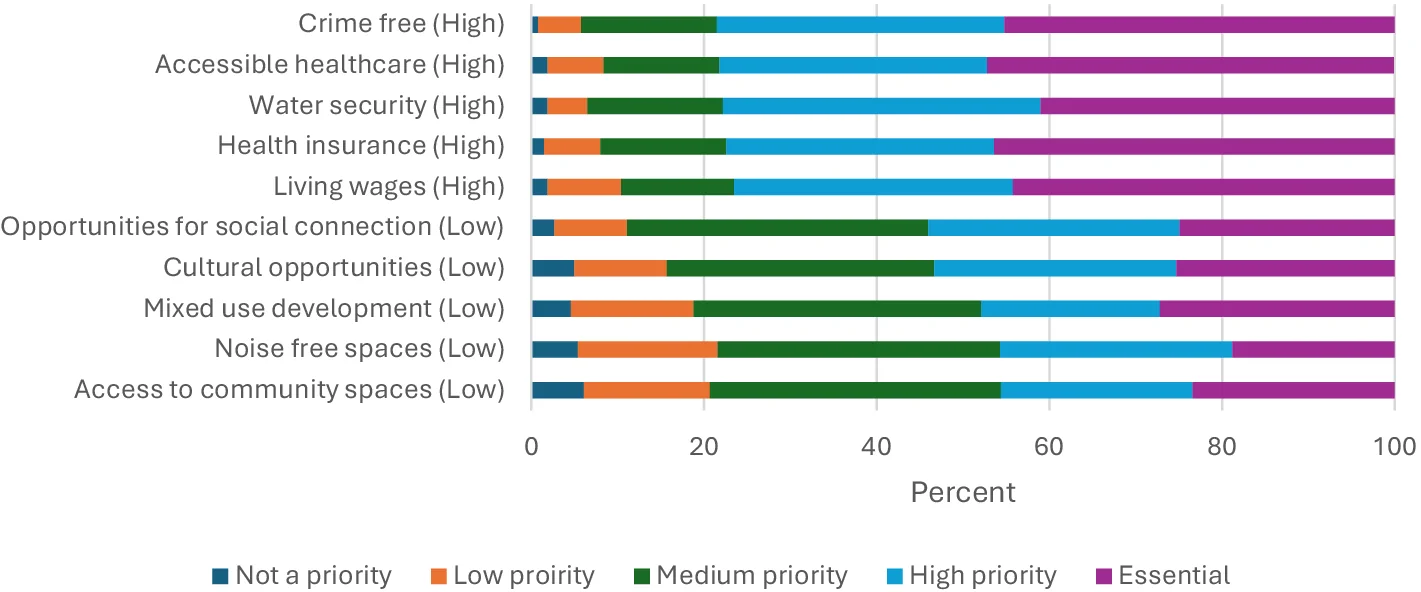
Response distribution for highest and lowest ranked community health priorities.

Response distributions were calculated for all community health priority questions. To identify the highest and lowest ranked priorities, the “Essential” and “High Priority” categories were combined, and items were ranked from highest to lowest. The five highest ranked health priorities were: 1. Communities free from crime and violence, 2. Accessible healthcare, 3. Water security, 4. Health insurance, and 5. Living wages. The lowest ranked health priorities were: 1. Access to community space, 2. Noise free space, 3. Mixed use development, 4. Cultural opportunities, and 5. Opportunities for social connection. Figure 2 presents the response distributions for the highest and lowest ranked priorities.

### Community assets and desired changes

**Figure 3:**
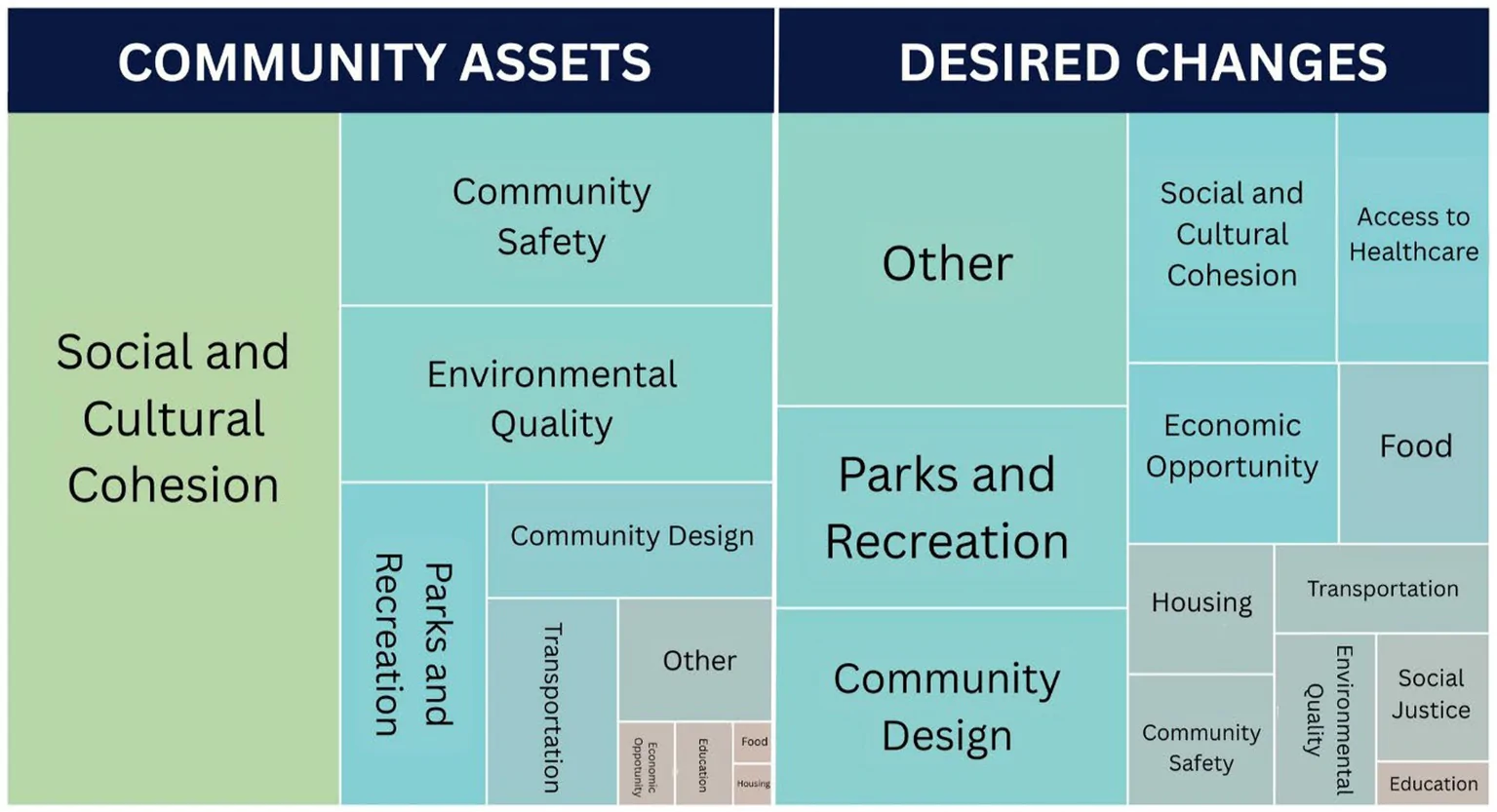
Tree map depicting themes related to community assets and desired changes.

Figure 3 depicts a tree map of qualitative themes related to the questions “What do you like most about living in your community?” and “What would you like to change about your community?” The area of each rectangle in the tree map is proportional to the number of times it was referenced by participants. Participants overwhelming recognized “social and cultural cohesion” as a community asset while “Parks and recreation” and “community design” were most frequently cited as areas for change in the community. In most cases, themes were identified as both community assets and desired changes; however, they differed in value (i.e., size of rectangle) across questions. Notably, “Access to healthcare” is only identified as something participants would like to change about their community.

### Community partner feedback

Overall, recruitment and survey implementation were most easily managed at NCPHSD. Recruitment was primarily conducted by staff at Women, Infants, and Children (WIC) clinics, immunization sites, and at community events. Community partners indicated that participants were excited about the incentive and only occasionally encountered access issues to the gift card or survey, thus requiring assistance from front-line staff.

Representatives from Canyonlands and the RCBH expressed similar issues related to recruitment and survey dissemination. Both shared that the pervasive issue at certain sites was technological and internet literacy as a barrier for survey access. For example, a QR code on a flier was used as the screener survey access point, and many required assistance with navigation and survey completion. Additionally, many community members filled out the screener survey but had difficulty accessing or completing the full questionnaire. Additional issues presented in bilingual accessibility for certain communities, and community differences in trust of outside organizations informed hesitancy to participate.

Each community partner organization suggested incorporating either a mixed media approach (i.e., both online and paper survey options) or exclusively a paper option for the future. Additionally, participants often commented that they would have preferred to complete the survey when they were at the clinic. Further, there was some variability in reception of the e-gift card. While many participants appreciated the flexibility of the card to be used wherever they liked, others stated that they would have preferred it to be both a physical card and to a local retailer. Importantly, each representative disclosed their appreciation for the existing partnerships and expressed enthusiasm for future collaboration.

## Discussion

This study explored the feasibility of using CCM as a tool for identifying community health priorities across diverse, underserved regions in Arizona. In keeping with the methodological focus of this paper, we do not present CCM analytic findings here; instead, we reflect on the feasibility and process of integrating CCM into community-engaged research. Guided by the mission of the RCMI CECs, our approach aimed to enhance traditional participatory methods in public health by integrating a structured, quantitative technique capable of capturing shared cultural beliefs.

Participatory approaches such as Photovoice, Participatory Mapping, and Digital Storytelling empower communities to articulate lived experiences and define health priorities (22); however, these methods often lack a mechanism to systematically quantify consensus. CCM addresses this gap by offering a statistically grounded framework to identify shared beliefs and determine which individuals best represent them (6). By quantifying agreement across participants, CCM helps validate whether insights from participatory methods reflect widespread community views or isolated perspectives (14).

Additionally, CCM enables researchers to identify cultural variability within and across communities, offering insights into subgroup differences and informing tailored interventions. Its ability to estimate individual competence scores allows for the identification of community experts—those whose views most closely align with the consensus—who can serve as valuable partners in future initiatives, such as an RCMI community expert board (11, 13).

CCM has been applied across diverse areas of health research to quantify shared beliefs, illuminate sources of disagreement, and strengthen the cultural validity of interventions and evaluations. In clinical settings, CCM has been used to identify value differences among patients, residents, and faculty, informing targeted improvements in communication and workflow (25). In global health, Strong and White (12) demonstrate how using CCM can enhance program planning, implementation, and evaluation by identifying locally shared understandings of maternal health and care-seeking, thereby improving the cultural fit of interventions in low-resource settings.

CCM has also been used to examine shared explanatory models of chronic disease. Pellosmaa (26) applied CCM to assess consensus in beliefs about type II diabetes and hypertension, showing how illness representations shape self-regulation and management behaviors among patients. Similarly, Simpson (27) used CCM to evaluate cultural models of lymphatic filariasis in Haiti, demonstrating how shared and divergent beliefs can serve as indicators of cultural change and guide public health messaging and evaluation strategies. Collectively, these studies illustrate how CCM provides a rigorous empirical foundation for understanding cultural models that influence health behaviors, enabling researchers and practitioners to design interventions that are more culturally aligned, contextually grounded, and responsive to community realities.

### Interpretation of findings

While past research suggests the methodological complexity of CCM can deter researchers from applying it in large-scale public health studies (14), our findings demonstrate that CCM can be successfully integrated into community-engaged research, even in geographically and culturally diverse settings; though, recruitment and demographic data underscore the importance of tailored engagement strategies when working with underserved rural and border communities. Although 62% of screened individuals were eligible, technological and internet literacy emerged as significant barriers to participation. These findings align with prior research on digital access disparities in rural populations and highlight the need for multimodal survey dissemination strategies (28). Community partner recommendations for paper-based or clinic-administered surveys further support this approach.

Participants prioritized structural determinants of health, ranking “communities free from crime and violence,” “accessible healthcare,” and “living wages” among the top five priorities—echoing national trends in rural health advocacy (29). While there was variation in how participants ranked health priorities, it is worth noting that all items were skewed toward the higher priority ratings. That is, there were no items that respondents on average indicated were not important to the health and wellbeing of their community.

Furthermore, qualitative analysis revealed the presence of similar themes across both “community assets” and “desired changes.” For instance, participants identified “Parks and recreation” as both a community asset and as something they’d like to change about their community. While this may seem counterintuitive, researchers believe this implies variability across communities and may indicate the potential of multiple cultural models. Notably, “access to healthcare” appeared solely as a desired change, suggesting this is an issue faced by all communities regardless of geography or culture.

Our findings also align with and extend prior research demonstrating how cultural models shape health priorities and engagement with public health systems. For example, earlier CCM studies have shown that cultural beliefs about infant feeding (8) and the interplay between culture, socioeconomic status, and health (9) can meaningfully influence health behaviors—paralleling our participants’ emphasis on structural determinants such as safety, healthcare access, and economic stability. The potential presence of multiple, coexisting cultural models in our data echoes work by Atran et al. (10), who found that cultural models often diverge across subgroups even within the same geographic region.

Similarly, the digital literacy and access barriers identified by our community partners reinforce broader evidence of structural inequities in rural and underserved communities (20), suggesting that methodological tools like CCM must be paired with flexible, community-driven implementation strategies to ensure equitable participation. By demonstrating that CCM can be integrated into community-engaged research despite these contextual challenges, our study contributes to a growing body of work illustrating how consensus-based approaches can surface both shared priorities and the structural constraints that shape how communities articulate and act on those priorities.

### Limitations

This study faced several limitations. First, a temporary funding freeze curtailed data collection in two planned regions, potentially limiting geographic representation. Second, compared to population averages in rural Arizona communities (30), our sample was disproportionately female and lacked racial diversity, which may affect the generalizability of findings. Of particular concern is the absence of representation from Native communities residing on Native lands, as IRB restrictions prevented their inclusion in the present study. Finally, technological barriers also impacted survey completion, particularly in communities with limited internet access or digital literacy.

### Future research

Working with safety net and community-based clinics helped expedite recruitment and facilitated the identification of underserved and rural participants. However, because recruitment occurred in clinical settings, the sample may be biased as women are more likely than men to seek primary care (31, 32). Future research in this area may benefit from recruitment strategies that target men and racial minorities, including partnering with community-based organizations that are not clinically focused.

Future analyzes will focus on using CCM to identify cultural models of community health priorities by geographic, demographic, and political affiliation. These analyzes fall outside the scope of the present feasibility manuscript and will be disseminated in a subsequent publication. These insights will inform future SHERC initiatives, including calls for proposals that reflect community-defined priorities. Additionally, researchers will collaborate with community partners to disseminate key findings to their community members and local decision makers. To improve generalizability of findings, future research needs to focus on conducting CCM with Native communities residing on Native lands.

## Conclusion

The present study demonstrates the feasibility of integrating CCM into community-engaged public health research and contributes to a growing body of literature advocating for the integration of anthropological methods into public health policy development and implementation (25, 33, 34). Because CCM mathematically identifies shared cultural beliefs, quantifies individual competence, works with small samples, and avoids imposing researcher-defined assumptions, it offers meaningful advantages compared to standard survey methods, interviews, and focus groups for identifying culturally grounded health priorities across diverse, underserved regions. By pairing CCM with participatory approaches and local partnership structures, researchers can capture both shared priorities and the contextual barriers—such as digital literacy challenges and structural inequities—that shape community participation and health concerns. These findings echo prior research showing that cultural models influence health behaviors and vary across subgroups (8–10), while also extending this work by illustrating how CCM can be operationalized within real-world public health systems. Additionally, this work aligns with the NIH’s Cultural Framework for Health (15), which calls for methods capable of measuring and integrating culture into health research and intervention design. As public health systems increasingly prioritize equity, inclusion, and community-defined priorities, CCM offers a rigorous and adaptable tool for elevating local knowledge, validating insights from participatory methods, and informing policy and program decisions that reflect the lived realities of rural, border, and underserved communities.

## Data Availability

The raw data supporting the conclusions of this article will be made available by the authors, without undue reservation.
